# Synergistic Effect of Atmospheric-pressure Plasma and TiO_2_ Photocatalysis on Inactivation of *Escherichia coli* Cells in Aqueous Media

**DOI:** 10.1038/srep39552

**Published:** 2016-12-22

**Authors:** Renwu Zhou, Rusen Zhou, Xianhui Zhang, Jiangwei Li, Xingquan Wang, Qiang Chen, Size Yang, Zhong Chen, Kateryna Bazaka, Kostya (Ken) Ostrikov

**Affiliations:** 1 Fujian Key Laboratory for Plasma and Magnetic Resonance, School of Physics Science and Technology, Xiamen University, Xiamen 361005, China; 2 School of Chemistry, Physics and Mechanical Engineering, Queensland University of Technology, Brisbane, Queensland 4000, Australia; 3 Department of Chemical and Biochemical Engineering, College of Chemistry and Chemical Engineering, Xiamen University, Xiamen 361005, China; 4 ShenZhen Research Institute of Xiamen University, Shenzhen 518000, China; 5 School of Physics and Electronic Information, Institute of Optoelectronic Materials and Technology, Gannan Normal University, Ganzhou 341000, China; 6 CSIRO-QUT Joint Sustainable processes and Devices Laboratory, Commonwealth Scientific and Industrial Research Organisation, P.O.Box 218, Lindfield, NSW 2070, Australia

## Abstract

Atmospheric-pressure plasma and TiO_2_ photocatalysis have been widely investigated separately for the management and reduction of microorganisms in aqueous solutions. In this paper, the two methods were combined in order to achieve a more profound understanding of their interactions in disinfection of water contaminated by *Escherichia coli*. Under water discharges carried out by microplasma jet arrays can result in a rapid inactivation of *E. coli* cells. The inactivation efficiency is largely dependent on the feed gases used, the plasma treatment time, and the discharge power. Compared to atmospheric-pressure N_2_, He and air microplasma arrays, O_2_ microplasma had the highest activity against *E. coli* cells in aqueous solution, and showed >99.9% bacterial inactivation efficiency within 4 min. Addition of TiO_2_ photocatalytic film to the plasma discharge reactor significantly enhanced the inactivation efficiency of the O_2_ microplasma system, decreasing the time required to achieve 99.9% killing of *E. coli* cells to 1 min. This may be attributed to the enhancement of ROS generation due to high catalytic activity and stability of the TiO_2_ photocatalyst in the combined plasma-TiO_2_ systems. Present work demonstrated the synergistic effect of the two agents, which can be correlated in order to maximize treatment efficiency.

Non-equilibrium low temperature plasma inactivation of microorganisms has attracted increased attention in recent years due to its efficacy against a wide range of bacteria and fungi^1^,^2^. Among plasma-generated effects, highly-reactive chemical species, in particular reactive oxygen (ROS) and nitrogen species (RNS), are generally considered to be responsible for the observed antimicrobial efficacy^3^. To date, most of the studies have focused on the activity of species generated in the gas phase, however, the lifetime of chemical species generated in the gas phase is relatively short, as they are quenched rapidly during their frequent collisions with molecules or other species. High reactivity and short lifetime also means that only a fraction of the species generated during plasma treatment are able to penetrate the gas−liquid interface to reach the intended target^4^, potentially limiting the efficiency of gas phase plasma inactivation against microorganisms in a moist environment or in bulk liquids.

Killing efficiency can be enhanced by circulating bacterial suspension to facilitate the exposure of microorganisms to the species generated at the gas−liquid interface^5^, however this may not be feasible in some treatment systems. Alternatively, sufficiently high concentrations of reactive species can be attained by generating atmospheric-pressure plasma directly in water, where the short-lived species can be formed in the near proximity of the target microorganisms. Higher chemical reaction rates of thus-generated species translate to significantly improved killing efficacy, as demonstrated by efficient inactivation of *Bacillus subtilis* spores using a direct-current, cold atmospheric-pressure air plasma microjet generated in water^6^. Excellent plasma stability and extremely efficient killing of *P. fluorescens* cells within 5 min were reported for air microplasma produced in aqueous media by hollow-fiber based microplasma jets^7^.

Further enhancement of antimicrobial efficacy of plasma can be achieved via synergistic action of plasma-generated effects and conventional chemical drugs or biologically-active nanomaterials, where, in addition to direct generation of oxidative species, the multi-modal catalytic activity of plasma enhances chemical and biological reactivity of these antimicrobial agents. The nature of catalytic activity of plasma is complex^8^,^9^. In addition to plasma-initiated chemical effects^2^,^3^, physical effects that occur in electrical discharges in solution, such as strong electric fields, thermal effects, UV radiation, ultrasound and shock wave play an important role in facilitating certain types of chemical reactions. As well as promoting chemical reactions, these effects may also directly contribute to the killing of the microorganisms, e.g. by changing the transport across the cell membrane, or declumping the cells, however their individual contributions to the overall biological activity of plasma have received relatively little attention^10^. In view of this, developing a combined technology to utilize both chemical and physical effects derived from plasma would be imperative and meaningful.

This paper explores the synergistic effects that may arise from coupling plasma with another advanced oxidation process, i.e. TiO_2_ photocatalysis, with specific focus on enhanced generation of oxidative species. Owing to its availability, low cost, chemical stability, and minimal toxicity^11^,^12^, TiO_2_ photocatalysis has emerged as a nonhazardous alternative to traditional chemical disinfection methods for inactivation of a wide range of pathogenic microorganisms. As shown in Fig. 1, the surface of TiO_2_ (particles or films) can be excited with light of wavelength shorter than 385 nm. The generated holes (h^+^) and electrons (e^−^) can react with H_2_O molecules to produce numerous reactive species (O_2_·^−^, OH), which can be efficiently used for microorganism inactivation^13^,^14^, water treatment^15^,^16^,^17^, air purification^18^, and cancer cell treatment^19^. As the generation of species is restricted by the broad band gap (> 3 eV) and low quantum efficiency of anatase TiO_2_, significant research efforts have been made to enhance the efficiency of the TiO_2_ catalyst by means of doping with various metals, e.g. Cu^20^ or Ag^21^, non-metals, e.g. carbon materials^22^,^23^, and narrow band semiconductors, such as V_2_O_5_^24^.

Highly-reactive plasma environment may provide an alternative mean for the enhancement of the TiO_2_ catalyst efficiency. By adjusting the processing parameters and treatment conditions, plasma can be modulated to preferentially generate metastables, ions, radicals, or (V)UV photons. As such, the high voltage discharge zone can serve as an effective source of (V)UV photons for TiO_2_ photocatalysis, generating a sufficient number of oxidative species to effectively inactivate *Bacillus subtilis* spores^25^. Furthermore, plasma-generated strong electric field may prevent recombination of electrons with holes on the surface of TiO_2_, thus improving the quantum efficiency of photocatalysis^23^. Moreover, ultrasound and shock waves generated in electrical discharge process can clean the TiO_2_ surface and enhance the mass transport of the reactants to the solid surface, thereby facilitating participation of more active catalyst sites in the heterogeneous catalytic reactions^26^.

The formation of strong electric fields, thermal effects, UV radiation, ultrasound and shock waves depend strongly upon the discharge parameters, including gas composition. Therefore, in this study, we investigated the antibacterial efficiency of plasma/TiO_2_ photocatalysis system (Fig. 2), where microplasma were generated in four types of gases, namely N_2_, He, air and O_2_, known to produce vastly different biological effects. The effect of treatment system geometry and energy of the discharge on the antibacterial efficacy was investigated. The inactivation efficiency of *Escherichia coli* cells, evaluated via Colony-Forming Units (CFU) counts on petri dishes, was correlated with the concentrations of key reactive oxygen species (ROS), i.e. hydrogen peroxide (H_2_O_2_), ozone (O_3_), hydroxyl radicals (·OH), to understand the mechanisms that may govern the observed antibacterial efficacy.

## Results

### Chemical reaction rate model and inactivation efficiency

Plasma-generated species, such as ·O, ·OH, H_2_O_2_ or O_3_, play an important role in microorganism inactivation and cell death^25^. Different empirical kinetic models can be used to elucidate the observed bacterial inactivation process. Among them, pseudo-first-order kinetics has been previously shown to produce best fit to the experimentally obtained data for the inactivation of microorganisms by advanced oxidation processes (AOPs). Given that the kinetic rate is widely applied to evaluate different disinfection processes, the process of *E. coli* disinfection in this study was fitted with CW model expressed by Eq. 1 23,27,28:


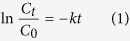


where *C*_*0*_ and *C*_*t*_ are the respective concentration of live *E. coli* cells at the start (t = 0) and the end of treatment (t), respectively, and *k* is the kinetic rate.

### Effect of gas chemistry and treatment time on bacterial inactivation

The sensitivity of *E. coli* cells to different types of plasma treatment is presented in Fig. 3. The efficacy of the treatment showed strong dependence on both the treatment time and the type of gas being used. Under the same experimental conditions (4.5 kV, 2 SLM flow rate, 10^6^ CFU/ml), the highest inactivation efficiency was observed for O_2_ microplasma, followed by air microplasma, He microplasma and N_2_ microplasma in the order of decreasing efficacy. Increasing exposure time significantly enhanced the decontamination effect of the treatment, with 4 min and 5 min being sufficient to achieve >99% bacterial inactivation using O_2_ and air plasma, respectively.

The experimentally obtained inactivation data were then evaluated using a kinetic model. To calculate the pseudo-first-order rate constant associated with the disinfection kinetics, a linear regression was performed on the data plotted in Fig. 3(e). Figure 3(e) shows the ln(*C*_*t*_*/C*_*0*_) values measured as a function of N_2_, He, air, or O_2_ plasma treatment time and fitted theoretically by using Eq. 1. As expected, the ln(*C*_*t*_*/C*_*0*_) values decreased with increasing the treatment time. The calculated theoretical kinetic rates of *k*(N_2_) = 0.009 s^−1^, *k*(He) = 0.022 s^−1^, *k*(air) = 0.052 s^−1^ and *k*(O_2_) = 0.076 s^−1^ were in good agreement with the measured values (*R*^2^ > 0.995 for all feed gases). This indicates that the plasma-generated ROS may play a crucial role in killing the microorganisms, with the efficacy of the treatment directly linked to the density of oxidants generated inside the microplasma. Among these four types of microplasma arrays, the O_2_ microplasma array was the most efficient in killing *E. coli* cells in water, which can be attributed to its relatively high density of ROS.

### Effect of TiO_2_ photocatalysis on the antibacterial performance of O_2_ microplasma

Identified as the most promising type of plasma for bacterial inactivation, the O_2_ discharge was then selected to investigate the effect of TiO_2_ photocatalysis on the antibacterial performance of the plasma in water system. Figure 4 shows the comparison between the ln(*C*_*t*_*/C*_*0*_) values measured in *E. coli* inactivation experiments performed with the O_2_ plasma-SD, CTD, and PTD systems and fitted theoretically by Eq. 1. Kinetic rates (*k*) and correlation coefficients (R^2^) derived from the fittings are summarized in Table 1. The theoretical values are in agreement with the measured values, with correlation coefficients as high as 0.995. Increasing the energy of discharge (*V*_P_) positively contributed to the enhancement of inactivation efficiency of the treatment across all three discharge systems (SD, CTD and PTD).

Furthermore, integration of TiO_2_ film into the treatment system greatly improved its ability to inactivate *E. coli* cells independent of input voltages. CTD configuration was particularly effective, showing consistently higher inactivation rate and shorter treatment times. *E. coli* cells with the concentration of 10^6^ CFU/mL were completely inactivated by O_2_ microplasma in the CTD system at *V*_P_ = 6.0 kV within the treatment time of 1.0 min. Such increase in activity was attributed to a higher concentration of ROS generated in aqueous solution by the TiO_2_ film photocatalyst and the O_2_ microplasma.

### Concentration of reactive oxygen species (ROS) under different plasma conditions

Table 2 shows changes in ROS (H_2_O_2_, O_3_ and ·OH) concentration in aqueous solutions treated with N_2_, He, air and O_2_ plasma as a function of treatment time. Longer treatment times resulted in a significant increase in the concentration of all ROS. The concentration of H_2_O_2_ in the distilled water attained levels of 1.463, 2.036, 5.644 and 14.013 mg/L after 5 min treatment with N_2_, He, air and O_2_ plasma discharges, respectively, at 4.5 kV. This was attributed to the high electron density, energy of the plasma and long lifetime of the excited species that facilitate the energy transfer between the excited plasma species and water molecules, leading to H_2_O_2_ formation (*e^−^ + H_2_O → ·H + ·OH + e^−^, ·OH + ·OH → H_2_O_2_)^2^. In addition, the dissolved zone (O_3_), acting as ROS, has been considered to be very effective against a wide range of microorganisms^3^. Compared with that in the air and O_2_ plasma treated solutions, the generated ozone concentration was significantly lower in solutions treated with either He or N_2_ microplasma. The highest ozone concentration of approximately 0.758 mg/L was produced in the O_2_ microplasma system, followed by that in the air microplasma (at 0.700 mg/L), He microplasma (at 0.080 mg/L), and N_2_ microplasma (at 0.047 mg/L).

More importantly, the concentration of OH radicals, a species widely considered to be among the most important agents in the bacterial inactivation process^25^,^29^, showed a remarkable increase with the treatment time, attributed to electron impact dissociation of water^30^. Treatment with O_2_ microplasma showed the highest ·OH concentration (3.998 mg/L) after 5 min, with slightly lower values obtained in solutions treated with air plasma (at 3.683 mg/L). On the other hand, ·OH concentration in solutions treated by He and N_2_ microplasma were relatively low (at 0.363 mg/L and 0.247 mg/L, respectively). The ROS concentration in the plasma-treated solutions is well related with the rate of killing of *E. coli* cells in water. The higher the ROS concentration is, the higher the sterilization efficiency would be.

Addition of TiO_2_ photocatalyst to O_2_ plasma system resulted in a notable increase in the concentration of H_2_O_2_, O_3_ and ·OH, with the results for different plasma discharge systems (SD, CTD and PTD) summarized in Table 3. Similar to the single-discharge system (without TiO_2_ photocatalyst), the respective concentrations of H_2_O_2_, O_3_ and ·OH increased with treatment duration. Increasing input voltage also resulted in a notable increase in the concentration of ROS in all systems. Interestingly, an increase of input voltage from 3 kV to 4.5 kV led to a multi-fold increase in the concentration of all ROS, whereas subsequent increase of input voltage to 6 kV only produced a modest increase in the ROS concentration.

In terms of system configuration, the highest concentrations of H_2_O_2_, O_3_ and ·OH were produced in the CTD system, reaching the levels of 17.985, 0.989 and 8.098 mg/L, respectively. Slightly lower values of 16.909, 0.865 and 6.909 mg/L, respectively, were attained using the PTD system, with the lowest respective levels of 15.494, 0.837 and 5.570 mg/L measured in the solution treated with SD system. These results indicate that the concentration of H_2_O_2_, O_3_ and ·OH in plasma-treated solution was significantly related to the TiO_2_ photocatalytic activity, and the synergistic effect between the TiO_2_ photocatalyst and O_2_ plasma may be optimized by changing the configuration of the treatment system. Furthermore, since the sterilization efficiency under 4.5 kV discharge in CTD and PTD was similar to that at the higher input voltage, 4.5 kV was recommended as the optimum discharge voltage in terms of energy consumption.

### Activity and stability of TiO_2_ films in O_2_ plasma system

*E. coli* inactivation efficiency of an original TiO_2_ film was compared with that of a ‘used’ TiO_2_ film, the latter being used for six treatments prior to this measurement. Figure 5 shows the performance of CTD, and PTD O_2_ plasma systems containing either the original or used TiO_2_ film when operated at 4.5 kV. In the CTD system containing the used TiO_2_ film, the inactivation efficiency of *E. coli* was similar to that fitted with an original TiO_2_ film. In the PTD system, however, the *E. coli* inactivation efficiency decreased when the used TiO_2_ film was employed, although it was still higher than that in the SD system. The results indicate that a TiO_2_ film deployed in the CTD system retained high photocatalytic activity over a longer period of use, whereas its activity was partly lost in the PTD system. This can probably be attributed to the change of TiO_2_ phase film during discharge channel formation in the PTD system but not in the CTD system, which was proven with the experimental XRD data.

### Structural characterization of TiO_2_ films before and after O_2_ plasma discharge

Figure 6 shows the XRD patterns of TiO_2_ films before and after their repeated use in the O_2_ plasma treatment system operated at 4.5 kV. After exposure to O_2_ discharge in the CTD system, the TiO_2_ film retained the same anatase phase diffraction peaks as the original sample, which also confirmed that the TiO_2_ film had not been changed during plasma treatment. In the PTD system, however, the phase of TiO_2_ had been altered since several peaks representing the rutile phase were observed in the diffraction pattern. In the PTD system, the plate-like TiO_2_ film was attached to the stainless steel surface acting as the ground electrode, the temperature of which would increase rapidly in the discharge process, leading to the TiO_2_ crystal structure transfer from anatase phase to rutile phase^19^,^31^. The rutile TiO_2_ has been previously demonstrated to possess lower catalytic activity than the anatase counterpart^11^,^31^, which may account for the decreased photocatalytic activity and inactivation efficiency of the used TiO_2_ in the PTD system.

## Discussion

Plasma-generated ROS, such as singlet oxygen (O radical), hydrogen peroxide (H_2_O_2_), ozone (O_3_) and hydroxyl radical (·OH), are generally considered to play significant roles in sterilization and decontamination of surfaces and liquids. When generated in gas phase for the treatment of pathogens in liquid phase, these species need to cross the gas−liquid interface in order to reach the intended target. This presents a considerable challenge, since the characteristic time of reaction between the O radical and pure water is *τ*_1_ = 0.02 s, the diffusion coefficient in water is *D*_1_ = 2 × 10^−9^m^2^s^−1^ and thus the estimated diffusion distance is 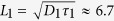 μm^32^,^33^. Therefore, it is difficult for these highly reactive radicals to penetrate the gas−liquid interface (several μm to hundreds of μm) or diffuse into the solution within their short lifetime during plasma treatment^33^,^34^. As for H_2_O_2_ and O_3_, they both have the same bacterial inactivation mechanism, reacting with H_2_O molecules to form OH radicals (O_3_ + H_2_O → 2OH + O_2_ and 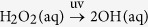), although the oxidation-reduction potential of H_2_O_2_ (acid, 1.776 V) is much lower than the corresponding value for O_3_ (2.07 V)^35^,^36^. By generating species in the close proximity to the intended microbial targets, i.e. by creating a discharge in the solution itself, it is possible to significantly enhance the decontamination efficacy of plasma treatment.

Despite significant progress in our understanding of the electrical discharges in aqueous solutions, many chemical and physical aspects of this process remain poorly understood. This is in part due to the complexity of the plasma-generated hydrodynamic effects, such as high liquid inhomogeneity due to complex nonlinear behavior which may affect the electric field, discontinuous liquid-vapor boundaries due to liquid vaporization, increased solution conductivity due to plasma generation of charged species, and increase in molecular density in the vicinity of the high voltage electrode. These effects will also depend on the operating parameters of the plasma, such as applied energy or gas chemical composition.

Our results show that among the N_2_, He, air, and O_2_ microplasma arrays, the O_2_ microplasma is the most effective in killing *E. coli* cells suspended in water, at least in part due to the relatively higher concentration of the ROS generated by this plasma in the treated solution. Once generated in the gas phase, these ROS species can immediately attack *E. coli* cells at the liquid/gas interface, or they may dissolve into the water, reaching the cells further away from the discharge. The increase in the inactivation efficiency with the treatment time is well explained by the chemical reaction rate model.

Depending on the voltage and the chemistry of the gas used to produce the plasma, as well as the duration of exposure, plasma may trigger either the physical disruption of bacteria or a cascade of biochemical reactions leading to bacterial death^3^,^29^. The Lissajous figures of the N_2_, He, air, and O_2_ microplasma arrays indicate that the discharge power showed little dependence on the chemistry of the feed gas used. However, the discharge power was found to increase rapidly, from 12W to 50W, as the *V*_*P*_ increased from 3.5 to 6.0 kV. Figure 7 shows the total ROS concentration in solutions treated in SD, PTD and CTD O_2_ plasma systems as a function of plasma discharge power. The CTD O_2_ microplasma system was found to generate the highest concentration of ROS under every discharge power, generating ∼27.5 mg/L of ROS at 50W, compared to ∼21.8 mg/L and ∼24.7 mg/L generated at the same discharge power using SD and PTD systems, respectively. Lower efficiency of PTD system may be attributed to the partial transition of TiO_2_ from the anatase into less catalytically-active rutile phase as a result of plasma-generated heating of the ground electrode onto which the TiO_2_ film was mounted.

In all systems tested, a strong positive correlation between the sterilization efficiency and the ROS concentration was demonstrated, suggesting a possible avenue for process optimization, for instance, through fast and efficient transport of plasma-generated species to the target organisms^2^,^35^,^37^. Generally, cell membranes containing unsaturated fatty acids and protein molecules can sustain significant damage from the strong oxidizing effects of ROS^6^, interfering with the ability of the cell membrane to effectively transport ions and polar compounds. Moreover, the plasma sheath can be formed in vicinity of *E. coli* cells located at the plasma/liquid interface, leading to charge accumulation on the bacterial surface^36^. The electrostatic force caused by charge accumulation on the outer surface of the cell membrane could overcome the tensile of the membrane and cause its rupture^29^,^36^.

Our study also showed the synergistic activity that arises as a result of combining two advanced oxidation processes, namely TiO_2_ photocatalysis and microplasma treatment. The improvement in killing efficacy was attributed to enhanced generation of ROS, which was in line with previous studies that showed substantial increase in the relative emission intensities of OH (313 nm) and O (777 nm) radicals in distilled water (1.7 and 3.8 times, respectively) in plasma systems augmented with TiO_2_ photocatalytic layer^16^. Figure 8 shows the likely reaction mechanism in the TiO_2_-microplasma photocatalysis process in the CTD system. When the plasma is generated between the high-voltage electrodes and ground electrode, large amounts of UV photons and ozone are produced in the plasma-discharge zone. These plasma-generated photons reach the surface of the TiO_2_ film, inducing photocatalysis, resulting in the generation of more OH radicals, and other active species (e.g., H_2_O_2_ and ·O_2_^−^). In addition, ozone accepts electrons to produce ·O_3_^−^ radicals, from which OH radicals are generated by radical chain reactions. A series of photocatalytic redox reactions are presented in Eq. 2,3,4,5,6.


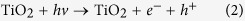



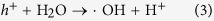



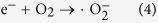



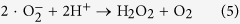



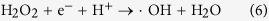


Active species (H_2_O_2_, O_3_, ·O_2_^−^ and ·OH) produced as a result of the aforementioned reactions in the plasma-enhanced TiO_2_ photocatalytic system can then be used to decontaminate polluted water, by directly killing pathogenic microorganisms, such as *E. coli* cells in this study, via oxidative stress, and by oxidizing the organic pollutants, such as organic dyes and phenolic derivatives, into smaller, less-hazardous molecules^15^,^16^,^17^,^38^. Depending on the chemistry of the pollutant or the species of the pathogen, it may be necessary to optimize the treatment system. For instance, in our study, highest efficiency was attained in the TiO_2_-microplasma combined systems in the CTD configuration fed with O_2_ gas. The presence of TiO_2_ provides an additional mechanism for bacterial inactivation, whereby pathogens located on TiO_2_ surface are subject to direct electron/hole exchange with the surface, affecting the cell membrane, which renders them more susceptible to oxidative damage by generated ROS. Once inside the cell, ROS can induce significant damage to intracellular components of the organism, including carbohydrates, lipids, proteins and nucleic acids, affecting cellular metabolism, and, at sufficiently high concentration, leading to cell death^14^,^19^. Importantly, our results show that the photocatalytic activity of TiO_2_ can be maintained over time, suggesting the proposed system may be used over relatively long periods of time without substantial loss in decontamination efficiency.

## Conclusion

In this study, the plasma inactivation of *E. coli* cells suspended in water was performed by using atmospheric-pressure N_2_, He, air, and O_2_ microplasma arrays. Compared to the N_2_ and He microplasma arrays, the air and O_2_ microplasma arrays are more effective in inactivating *E. coli* cells in water. Addition of TiO_2_ photocatalysis to the O_2_ microplasma system can lead to substantial improvements in the inactivation efficiency against *E. coli*, attributed primarily to the enhancement of ROS generation. System configuration, specifically the placement of TiO_2_ thin film, notably affected both the ROS generation and the longevity of the photocatalyst, with the highest inactivation efficiency and stability observed in the CTD system operated with O_2_ as the feed gas. The chemical reaction rate model provided good fit to the experimentally-obtained data, and may be used to quantitatively describe the efficiency of *E. coli* cell inactivation in single-discharge and plasma-TiO_2_ combined systems.

## Methods

**Figure 2:**
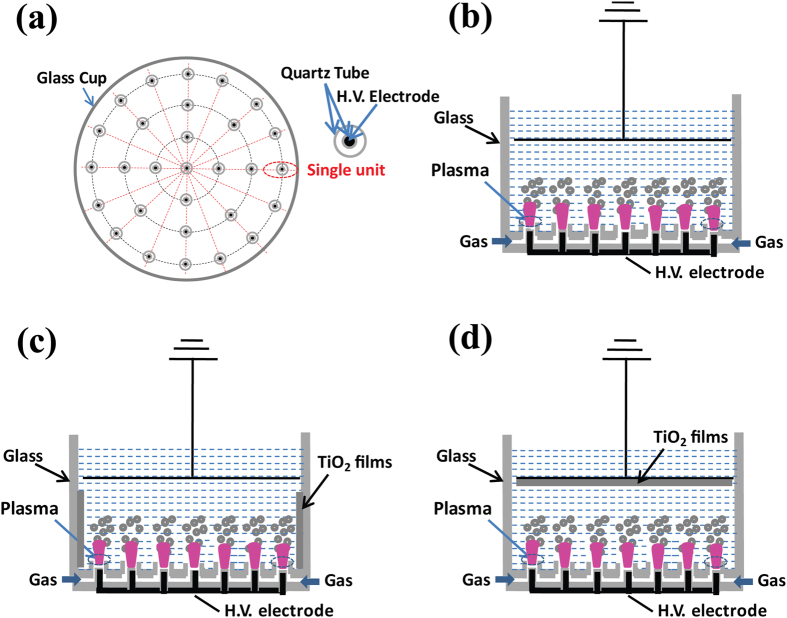
Schematic diagram of the well-aligned air microplasma jet array operating at atmospheric pressure. Top view of the microplasma device (**a**) and cross-sectional view of single-discharge system without TiO_2_ (SD) (**b**), SD system with a circular TiO_2_ film (CTD) (**c**), and SD system with a plate-like TiO_2_ film (PTD) (**d**).

**Figure 3:**
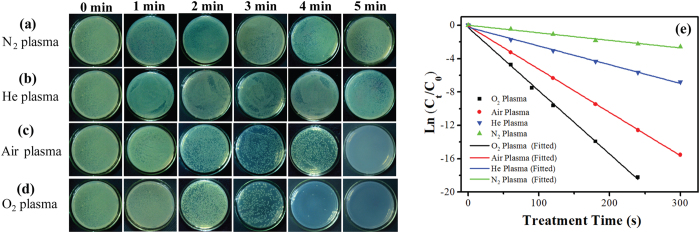
Viability of *E. coli* cells treated using microplasma arrays fed with (**a**) N_2_, (**b**) He, (**c**) air, and (**d**) O_2_ gases as a function of treatment time. The plasma treatments have been performed at *V*_P_ = 4.5 kV. (**e**) The comparison between empirical ln(*C*_*t*_*/C*_*0*_) and theoretical kinetic rate values (from Eq. 1).

**Figure 4:**
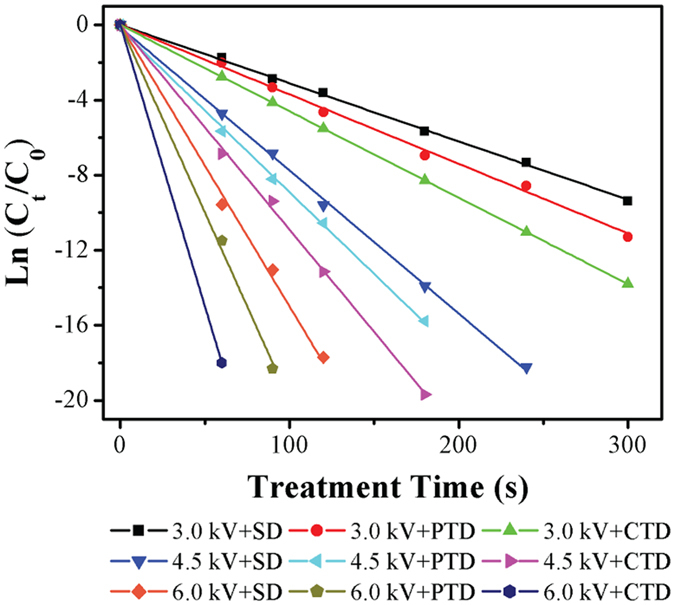
The comparison between the experimentally-obtained ln(*C*_*t*_*/C*_*0*_) and theoretically-predicted kinetic rate values (using Eq. 1) for different plasma discharge systems as a function of discharge energy (*V*_P_).

**Figure 5:**
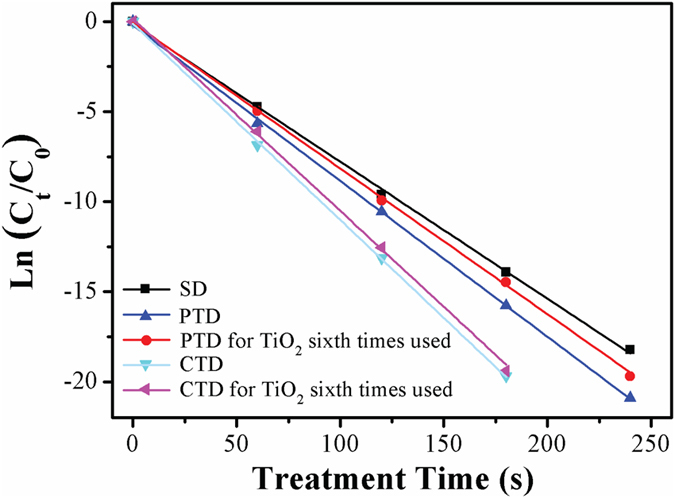
Comparison of the *E. coli* inactivation performance of CTD and PTD O_2_ plasma systems containing original or used TiO_2_ films. All O_2_ plasma treatments were performed at a *V*_*p*_ of 4.5 kV with different treatment time.

**Figure 6:**
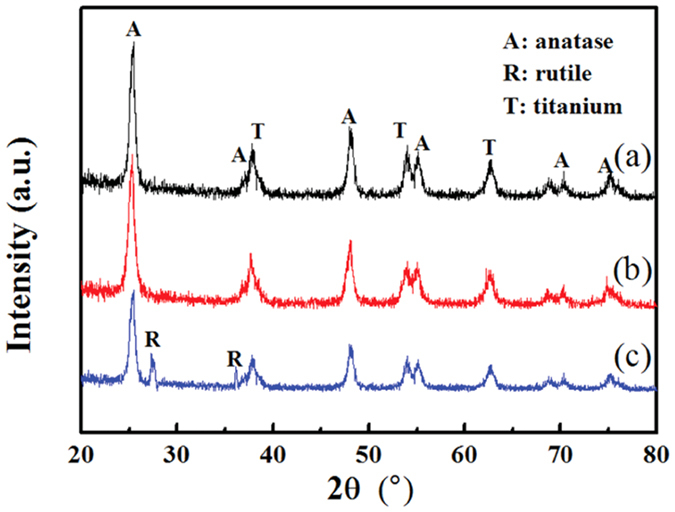
The XRD patterns of original (**a**) TiO_2_ films and those re-used for six times in CTD (**b**) and PTD (**c**) O_2_ plasma systems.

**Figure 7:**
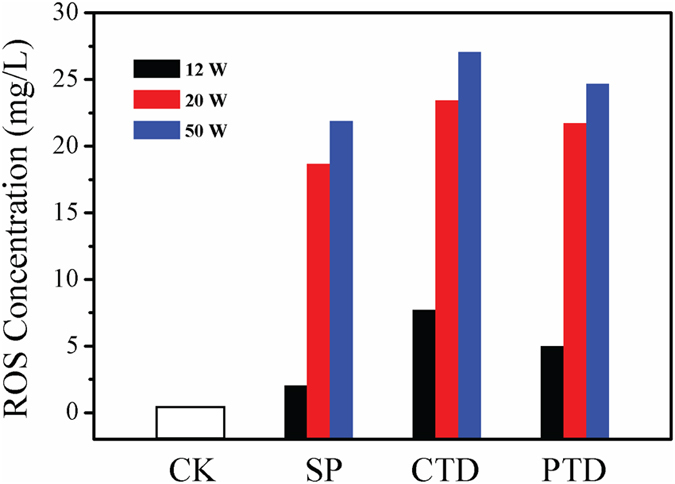
ROS concentrations in solutions treated with SD, PTD and CTD O_2_ plasma systems under different plasma discharge power of 12 W, 20 W and 50 W.

**Figure 8:**
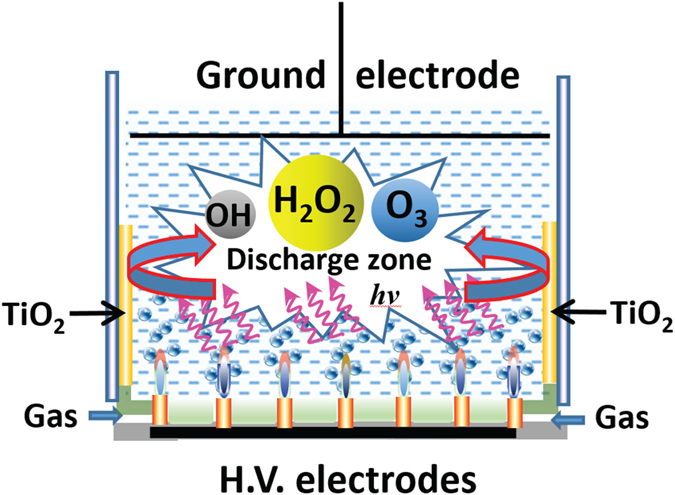
Reaction mechanism of plasma discharge combined with TiO_2_ films.

Atmospheric-pressure microplasma arrays were used to inactivate *E. coli* cells in aqueous media, as shown in Fig. 2^ ^7,39. Briefly, the plasma device consists of 29 microplasma jet units housed in a glass cup (inner diameter: 50 mm). Each discharge tube consists of three parts: (1) an inner electrode, (2) a quartz sleeve of inner electrode, and (3) an external quartz tube. The separation between the external quartz tube and the inner quartz sleeve is approximately 80 μm. When the gas passes through the separation distance, the plasma is produced by dielectric barrier discharge^37^. The feed gas, such as He, N_2_, air, or O_2_ is supplied into the discharge tubes from the bottom of the device at the flow rate of 2.0 standard liter per minute (SLM). A stainless steel ground electrode surface (Ø 30 mm) is positioned about 10 mm above the discharge tubes in the discharge reactor (as shown in Fig. 2).

Film of TiO_2_ nanotubes were prepared by an electrochemical method^40^. A 300-μm-thick titanium foil, acting as the anode, was cleaned with acetone for 15 min and then washed with distilled water for 15 min in an ultrasonic cleaner machine. One nickel plate was also immersed in electrolyte (0.08 mol/L C_2_H_2_O_4_·2H_2_O and 0.135 mol/L NH_4_F), acting as the cathode. The power supply was utilized to provide a DC output with the voltage of 20 V. After the 2 h electrochemical reaction, the anodized samples were calcined at 400 °C for 2 h in a muffle furnace.

Once prepared, TiO_2_ films were utilized in the plasma reactors in two configurations. In the first case, a circular TiO_2_ film was distributed around the inner surface of the discharge reactor cylinder (CTD), while in the second case, a plate-like TiO_2_ film was attached to the stainless steel ground electrode surface in the discharge reactor (PTD), as shown in Fig. 2(b) and (c), respectively. The power supply (TCP-2000K, Najing Suman Electronic, China) capable of supplying bipolar AC output with the peak voltage (*V*_P_) of 0–20 kV at an AC frequency of 9.0 kHz was used. The discharge power can be calculated by a Lissajous figure formed with the charges across this capacitor and the applied voltage across the discharge chamber.

A colony of *E. coli* was dispersed into approximately 200 ml of LB liquid medium (Tryptone: 2 g, Yeast Extract: 1 g, NaCl: 2 g, distilled water: 200 g, pH = 7.2) and was incubated on the shaking table (rotation speed: 200 rpm, 28 °C, 24 h) until a sufficient number of *E. coli* cells were obtained. Then, test solutions were prepared by inoculating sodium chloride solution (1% NaCl) to the concentration of 10^6^ CFU/ml. Then, a 100 ml aliquot of the test solution was treated by N_2_, He, air and O_2_ microplasma or these microplasma coupled with TiO_2_ films, with the treatment time ranging from 1 to 5 min. Control sample received no plasma treatment. After these treatments, 100 μl of the treated solution was inoculated on the standard petri dishes 9 cm in diameter containing approximately 15 ml of LB solid medium. The plates were then incubated for 24 h at 37 °C, at which stage colony counting was performed to estimate the survival rate of the treated bacteria.

The concentration of hydrogen peroxide in the plasma-treated water was obtained by color forming reactions and spectrophotometric measurements. When titanium oxysulfate (TiOSO_4_) reacted with H_2_O_2_, a yellow-colored complex (pertitanic acid) was formed and UV–Vis measurement was done at 407 nm to colorimetrically determine the concentration of H_2_O_2_ (TiO^2+^ + H_2_O_2_ → [TiO(H_2_O_2_)]^2+^)^41^. The concentration of dissolved ozone was determined by the indigo method^42^. For hydroxyl free radical detection, the salicylic acid (SA) was used as a selective trapping reagent of ·OH and the production of ·OH radicals was quantified by High-performance liquid chromatographic (HPLC) assay^43^.

## Additional Information

**How to cite this article**: Zhou, R. *et al*. Synergistic Effect of Atmospheric-pressure Plasma and TiO_2_ Photocatalysis on Inactivation of *Escherichia coli* Cells in Aqueous Media. *Sci. Rep.* **6**, 39552; doi: 10.1038/srep39552 (2016).

**Publisher's note:** Springer Nature remains neutral with regard to jurisdictional claims in published maps and institutional affiliations.

## Figures and Tables

**Figure 1:**
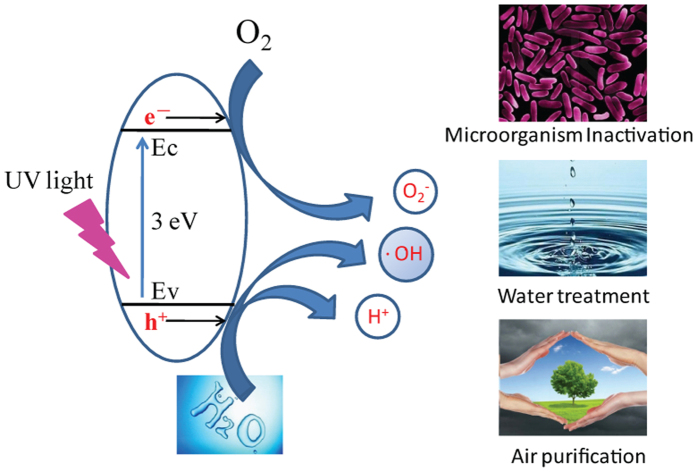
The mechanism of TiO_2_ photocatalytic activity for microorganism inactivation, water treatment and air purification.

**Table 1:** The kinetic rate at different *V*_P_ values and under different O_2_ plasma discharge systems.

Conditions	3.0 kV			4.5 kV			6.0 kV		
	SD	CTD	PTD	SD	CTD	PTD	SD	CTD	PTD
Kinetic rate (s^−1^)	0.031	0.046	0.037	0.076	0.110	0.089	0.150	0.300	0.200
R^2^	0.998	0.997	0.998	0.999	0.999	0.998	1	1	—

**Table 2:** Changes of ROS concentrations in aqueous solution with N_2_, He, air and O_2_ plasma treatments.

Conditions	N_2_ microplasma			He microplasma			air microplasma			O_2_ microplasma		
	1 min	3 min	5 min	1 min	3 min	5 min	1 min	3 min	5 min	1 min	3 min	5 min
H_2_O_2_ (mg/L)	0.352	0.863	1.463	0.782	1.349	2.036	1.325	3.892	5.644	3.947	8.764	14.013
O_3_ (mg/L)	0.022	0.036	0.047	0.033	0.057	0.080	0.536	0.612	0.700	0.620	0.678	0.758
·OH (mg/L)	0.076	0.119	0.247	0.092	0.163	0.363	0.903	2.093	3.683	1.445	2.517	3.998

**Table 3:** Changes of ROS concentrations in O_2_ plasma-treated solution under different plasma discharge systems.

Conditions	3.0 kV			4.5 kV			6.0 kV		
	SD	CTD	PTD	SD	CTD	PTD	SD	CTD	PTD
H_2_O_2_ (mg/L)	1.126	5.865	3.652	14.013	16.304	15.392	15.494	17.985	16.909
O_3_ (mg/L)	0.226	0. 560	0. 344	0.758	0.845	0.786	0.837	0.989	0.865
·OH (mg/L)	0.698	1.291	1.019	3.998	6.284	5.524	5.570	8.098	6.909

## References

[b1] Morfill G., Kong M. G. & Zimmermann J. Focus on plasma medicine. New J. Phys. 11, 115011 (2009).

[b2] Zhang X. H. . Atmospheric cold plasma jet for plant disease treatment. Appl. Phys. Lette. 104, 043702 (2014).

[b3] Lu X. P. . Reactive species in non-equilibrium atmospheric-pressure plasmas: Generation, transport, and biological effects. Phys. Rep. 630, 1–84 (2016).

[b4] Liu D. X. . Aqueous reactive species induced by a surface air discharge: Heterogeneous mass transfer and liquid chemistry pathways. Sci. Rep. 6, 23737 (2016). 27033381 10.1038/srep23737 PMC4817137

[b5] Du C. M. . The application of a non-thermal plasma generated by gas-liquid gliding arc discharge in sterilization. New J. Phys. 14, 013010 (2012).

[b6] Sun P. . Inactivation of Bacillus subtilis spores in water by a direct–current, cold atmospheric–pressure air plasma microjet. Plasma Process. Polym. 9, 157–164 (2012).

[b7] Zhang X. H. . Atmospheric-pressure air microplasma jets in aqueous media for the inactivation of *Pseudomonas fluorescens* cells. Phys. Plasmas 20, 053501 (2013).

[b8] Neyts E. C. . Plasma catalysis: synergistic effects at the nanoscale. Chem. Rev. 115, 13408–13446 (2015). 26619209 10.1021/acs.chemrev.5b00362

[b9] Bazaka K. . Sustainable life cycles of natural-precursor-derived nanocarbons. Chem. Rev. 116, 163–214 (2015). 26717047 10.1021/acs.chemrev.5b00566

[b10] Laroussi M. & Leipold F. Evaluation of the roles of reactive species, heat, and UV radiation in the inactivation of bacterial cells by air plasmas at atmospheric pressure. Int. J. Mass Spectrom. 233, 81–86 (2004).

[b11] Matsunaga T. . Photoelectrochemical sterilization of microbial cells by semiconductor powders. FEMS Microbiol. Lett. 29, 211–214 (1985).

[b12] Joost U. . Photocatalytic antibacterial activity of nano–TiO_2_ (anatase)–based thin films: Effects on *Escherichia coli* cells and fatty acids. J. Photoch. Photobio. B 142, 178–185 (2015). 10.1016/j.jphotobiol.2014.12.010 25545332

[b13] Dumitriu C. . Antibacterial efficiencies of TiO_2_ nanostructured layers prepared in organic viscous electrolytes. Appl. Surf. Sci. 341, 157–165 (2015).

[b14] Tallósy S. P. . Adhesion and inactivation of Gram-negative and Gram-positivebacteria on photoreactive TiO_2_/polymer and Ag–TiO2/polymer nanohybrid films. Appl. Surf. Sci. 371, 139–150 (2016).

[b15] Sahel K. . Photocatalytic decolorization of Remazol Black 5 (RB5) and Procion Red MX-5B-Isotherm of adsorption, kinetic of decolorization and mineralization. Appl. Catal. B 77, 100–109 (2007).

[b16] Wang H. . Enhanced generation of oxidative species and phenol degradation in a discharge plasma system coupled with TiO_2_ photocatalysis. Appl. Catal. B 83, 72–77 (2008).

[b17] Zaky A. M. & Chaplin B. P. Porous substoichiometric TiO2 anodes as reactive electrochemical membranes for water treatment. Environ. Sci. Technol. 47, 6554–6563 (2013). 23688192 10.1021/es401287e

[b18] Wang W. G. . Enhanced photocatalytic activity of hierarchical macro/mesoporous TiO_2_–graphene composites for photodegradation of acetone in air. Appl. Catal. B 119, 109–116 (2012).

[b19] Jukapli N. M. & Bagheri S. Recent developments on titania nanoparticle as photocatalytic cancer cells treatment. J. Photoch. Photobio. B. 163, 421–430 (2016). 10.1016/j.jphotobiol.2016.08.046 27639172

[b20] Leyland N. S. . Highly Efficient F, Cu doped TiO_2_ anti-bacterial visible light active photocatalytic coatings to combat hospital-acquired infections. Sci. Rep. 6, 24770 (2016). 27098010 10.1038/srep24770 PMC4838873

[b21] Zhang X. Y. . Antibacterial activity of single crystalline silver–doped anatase TiO_2_ nanowire arrays. Appl. Surf. Sci. 372, 139–144 (2016).

[b22] Rahimi R. . Visible light photocatalytic disinfection of *E. coli* with TiO_2_–graphene nanocomposite sensitized with tetrakis(4-carboxyphenyl) porphyrin. Appl. Surf. Sci. 355, 1098–1106 (2015).

[b23] Ouyang K. . Efficient photocatalytic disinfection of *Escherichia coli* O157:H7 using C_70_–TiO_2_ hybrid under visible light irradiation. Sci. Rep. 6, 25702 (2016). 27161821 10.1038/srep25702 PMC4861983

[b24] Sethi D. . Photocatalytic destruction of *Escherichia coli* in water by V_2_O_5_/TiO_2_. J. Photoch. Photobio. B 144, 68–74 (2015). 10.1016/j.jphotobiol.2015.02.003 25728225

[b25] Jung H. . Enhanced inactivation of bacterial spores by atmospheric pressure plasma with catalyst TiO_2_. Appl. Catal. B 93, 212–216 (2010).

[b26] Li J. . Research on decoloration of dye wastewater by combination of pulsed discharge plasma and TiO_2_ nanoparticles. Desalination 212, 123–128 (2007).

[b27] Alrousan D. M. A. . Solar photocatalytic disinfection of water with immobilised titanium dioxide in recirculating flow CPC reactors. Appl. Catal. B 128, 126–134 (2012).

[b28] Younas H. . Visible light photocatalytic water disinfection and its kinetics using Ag doped titania nanoparticles. Environ. Sci. Pollut. R. 21, 740–752 (2014). 10.1007/s11356-013-1980-7 23872896

[b29] Zhou R. W. . Reactive oxygen species in plasma against *E. coli* cells survival rate. Chinese Phys. B 24, 085201 (2015).

[b30] Chandana L., Reddy P. M. K. & Subrahmanyam C. Atmospheric pressure non-thermal plasma jet for the degradation of methylene blue in aqueous medium. Chem. Eng. J. 282, 116–122 (2015).

[b31] Yu J. C. . Enhancing effects of water content and ultrasonic irradiation on the photocatalytic activity of nano-sized TiO_2_ powders. J. Photoch. Photobio. A 148, 263–271 (2002).

[b32] Blauwhoff P. M. M., Versteeg G. F. & Swaaijvan W. P. M. A study on the reaction between CO_2_ and alkanolamines in aqueous solutions. Chem. Eng. Sci. 39, 207–225 (1984).

[b33] Shibata T. & Nishiyama H. Numerical study of chemical reactions in a surface microdischarge tube with mist flow based on experiment. J. Phys. D Appl. Phys. 47, 105203 (2014).

[b34] Samukawa S. . The 2012 plasma roadmap. J. Phys. D Appl. Phys. 45, 253001 (2012).

[b35] Zhou R. . Inactivation of *Escherichia coli* cells in aqueous solution by atmospheric-pressure N_2_, He, Air, and O_2_ Microplasmas. Appl. Environ. Microb. 81, 5257–5265 (2015). 10.1128/AEM.01287-15 PMC4495224 26025895

[b36] Song Y. . An atmospheric-pressure large-area diffuse surface dielectric barrier discharge used for disinfection application. IEEE T. Plasma Sci. 43, 821–827 (2015).

[b37] Zhang X. H. . Treatment of ribonucleoside solution with atmospheric-pressure plasma. Plasma Process. Polym. 13, 429–437 (2015).

[b38] Zhang Y. . Application of TiO_2_ nanotubes with pulsed plasma for phenol degradation. Chem. Eng. J. 215, 261–268 (2013).

[b39] Zhou R. W. . Interaction of atmospheric-pressure air microplasmas with amino acids as fundamental processes in aqueous solution. PLOS ONE 11, e0155584 (2016). 27183129 10.1371/journal.pone.0155584 PMC4868320

[b40] Lei L. . Fabrication of multi-non-metal-doped TiO_2_ nanotubes by anodization in mixed acid electrolyte. Mater. Res. Bull. 42, 2230–2236 (2007).

[b41] Jablonowski H. & von Woedtke T. Research on plasma medicine-relevant plasma-liquid interaction: What happened in the past five years? Clin. Plasma Med. 3, 42–52 (2015).

[b42] Clesceri L. S., Greenberg A. E. & Eaton A. D. Standard methods for the examination of water and wastewater, *American Public Health Association*, New York, 1998.

[b43] Diez L., Livertoux M. & Stark A. High-performance liquid chromatographic assay of hydroxyl free radical using salicylic acid hydroxylation during *in vitro* experiments involving thiols. J. Chromatog. B 763, 185–193 (2001). 10.1016/s0378-4347(01)00396-6 11710577

